# Novel methods to identify biologically relevant genes for leukemia and prostate cancer from gene expression profiles

**DOI:** 10.1186/1471-2164-11-274

**Published:** 2010-04-30

**Authors:** Austin H Chen, Yin-Wu Tsau, Ching-Heng Lin

**Affiliations:** 1 Department of Medical Informatics, Tzu Chi University, No.701, Sec. 3, Jhongyang Rd. Hualien City, Hualien County 97004, Taiwan; 2 Graduate Institute of Medical Informatics, Tzu Chi University, No.701, Sec. 3, Jhongyang Rd. Hualien City, Hualien County 97004, Taiwan

## Abstract

**Background:**

High-throughput microarray experiments now permit researchers to screen thousands of genes simultaneously and determine the different expression levels of genes in normal or cancerous tissues. In this paper, we address the challenge of selecting a relevant and manageable subset of genes from a large microarray dataset. Currently, most gene selection methods focus on identifying a set of genes that can further improve classification accuracy. Few or none of these small sets of genes, however, are biologically relevant (i.e. supported by medical evidence). To deal with this critical issue, we propose two novel methods that can identify biologically relevant genes concerning cancers.

**Results:**

In this paper, we propose two novel techniques, entitled random forest gene selection (RFGS) and support vector sampling technique (SVST). Compared with results from six other methods developed in this paper, we demonstrate experimentally that RFGS and SVST can identify more biologically relevant genes in patients with leukemia or prostate cancer. Among the top 25 genes selected using SVST method, 15 genes were biologically relevant genes in patients with leukemia and 13 genes were biologically relevant genes in patients with prostate cancer. Meanwhile, the RFGS method, while less effective than SVST, still identified an average of 9 biologically relevant genes in both leukemia and prostate cancers. In contrast to traditional statistical methods, which only identify less than 8 genes in patients with leukemia and less than 8 genes in patients with prostate cancer, our methods yield significantly better results.

**Conclusions:**

Our proposed SVST and RFGS methods are novel approaches that can identify a greater number of biologically relevant genes. These methods have been successfully applied to both leukemia and prostate cancers. Research in the fields of biology and medicine should benefit from the identification of biologically relevant genes by confirming recent discoveries in cancer research or suggesting new avenues for exploration.

## Background

The completion of the Human Genome Project (HGP) has been recognized as a great achievement in the study of biomedicine; the project not only provided comprehensive information on the human genome but also inspired new ways to study human diseases such as cancers. Concurrent with the advancement of the HGP, several high-throughput and rapid gene function analysis techniques were developed. Among them, microarray may be the most mature technique, and it has become a major data resource in gene function research [1-3]. Over the past few years, microarray-based gene expression profiling has proven to be a promising approach in predicting cancer classification and prognosis outcomes [4-6]. In most cases, cancer diagnosis depends on using a complex combination of clinical and histopathological data. However, it is often difficult or impossible to recognize tumor types in atypical instances [7]. To translate microarray data into functional physiological information, a set of genes with the maximum amount of information and a minimum amount of noise is needed. For example, diagnostic tests that measure the abundance of a given protein in serum may be derived from a small subset of biologically relevant genes.

In cancer classification, one of the reasons one may wish to select a minimum set of genes is to avoid an over-fitting problem caused by attempting to apply a large number of genes to a small number of samples. There are several statistical and machine learning techniques such as t-Test, k-nearest neighbors, clustering methods [8], self organizing maps (SOM) [9], genetic algorithm [10], back-propagation neural network [11-13], probabilistic neural network, decision tree [14], random forest [15], and support vector machines (SVM) [16,17] that have been applied in selecting informative genes. Although these methods can select smaller set of informative genes, only a small percentage of these so called "informative" genes are biologically relevant as proved by medical experiments. Our goal in this paper, therefore, is to best identify biologically relevant genes from a small set of genes using our proposed methods. We present a novel approach that addresses different considerations, including: (1) the identification of quality samples, (2) the selection of a small set of informative genes from these samples, (3) the comparison of these genes with medical literature, and (4) the interpretation of their biological relevance.

Prostate cancer and leukemia are very common cancers in the United States. In 2007 alone, approximately 24 800 new cases and 12 320 deaths among males were attributed to leukemia. Among males age 40 and below, leukemia is the most common fatal cancer. Meanwhile, 19 440 new cases and 9 470 deaths among females were attributed to leukemia, and it is the leading cause of cancer death among females below age 20. Acute lymphocytic leukemia (ALL) is the most common cancer in children age 14 and below. Prostate cancer, on the other hand, in 2007 accounted for almost 29% (218 890) of incidents in males. For men age 80 and older, prostate cancer is the second most common cause of cancer death. Based on cases diagnosed between 1996 and 2002, an estimated 91% of these new cases are expected to be diagnosed at the local or regional level, for which the 5-year relative survival rate approaches 100% [18,19]. Therefore, the identification of biologically relevant genes is of fundamental and practical interest. The examination of these genes may be useful in confirming recent discoveries in cancer research or suggesting new methods for exploration.

In this paper, we examine eight methods for identifying biologically relevant genes. Among them are six statistics methods [20,21] and two machine learning methods. The statistics methods include three parametric methods: Signal-to-noise ratio (SNR) [22-24], t-Test [23,25], and Least Significant Difference (LSD) [13,26]. They also include three nonparametric methods: Threshold Number of Misclassification (TNoM) [25], Minimum Distance to Modal Ranking (MDMR) [27,28], and Weighted Punishment on Overlap (WEPO) [29,30]. In addition to these six statistics methods, we propose two new methods using machine learning approaches: Random forest gene selection (RFGS) and Support Vector Sampling technique (SVST). For each one of these, we first introduce some underlying theory and the process of computation. Then, we apply these methods to both leukemia and prostate cancer datasets. We compare the top 25 genes identified by each method with those identified within current medical literature, thus pinpointing the biological genes most related to leukemia and prostate cancer. The results show that our proposed SVST method is significantly better than statistical methods for identifying relevant biological genes in leukemia and prostate cancer.

The remainder of this paper is organized as follows: Section 2 discusses the various statistics-based gene selection methods considered in the paper. Section 3 describes our two proposed machine learning methods. Section 4 describes the experiment results and discusses leukemia and prostate cancer. Finally, Section 5 presents the conclusions of our study.

## Statistics-Based Gene Selection Methods

Gene selection is widely used to select target genes in the diagnosis of cancers. One of the primary goals of gene selection is to avoid the over-fitting problems caused by the high dimensions and relatively small number of samples of microarray data. Theoretically, in cancer classification, only informative genes which are highly related to particular classes (or subtypes) should be selected [24]. In microarray data analysis, the challenge is to select informative genes that clearly differentiate the classes. Since the number of informative genes is very small compared to the total number of genes in each experiment, utilizing a better search technique is critical. We divide such techniques into two main categories: statistics-based methods and machine learning-based methods. In this section, we will discuss the statistics methods while addressing the machine learning-based methods in the next section.

The statistics methods rank or score the discriminability of each gene based on its own gene expression patterns. Both parametric and nonparametric approaches for estimations of discriminability have been proposed. The parametric estimation approach assesses the discriminability of genes using a variety of statistical analyses, including Signal-to-noise ratio (SNR), t-Test, and Least Significant Difference (LSD). Parametric estimation depends on exact expression levels and the number of replicate samples. The statistical criteria are based on the assumption that the data comes from some kind of distribution. Each parametric approach puts different weights on the variance and number of samples of the criteria. In this study, we use three parametric methods: Signal-to-noise ratio (SNR), t-Test, and Least Significant Difference (LSD). A gene is considered more informative if it possesses a larger corresponding score.

### Signal-to-Noise Ratio (SNR)

Each dataset consists of *m *samples and *n *genes. For each gene *g*_*i*_, we normalize the gene expression data by subtracting the mean (signal) and then dividing by the standard deviation of the expression value (noise). Every sample is labeled with {+ 1,-1} (e.g. normal or cancer). We use the following formula to calculate each gene's F score.

The *μ *and *σ *characters represent the mean and the standard deviation of samples in each class (either + 1 or -1) individually. We rank these genes by F score and then select the top 25 gene sets as the features.

### t-Test

The t-Test assesses whether the means of two groups are statistically different from each other. In microarray data analysis, the unpaired two-sample *t*-Test is often used since samples may be derived from different experiments and may have different distributions. We calculate the discriminative power of the *i*th gene using a t-Test,

where *M *+ and *M*- are the sample sizes and *μ *and *σ *are the respective mean and standard deviation of samples in each class (either + 1 or -1). We rank these genes with a T score and then select the top 25 gene sets as the features.

### Least Significant Difference (LSD)

Least Significant Difference, also called the Fisher criterion, is a classical measure to assess the degree of separation between two classes. It is a t-Test-like statistic. The score for gene *i *is defined as

where *μ *and *σ *are the respective mean and standard deviation of samples in each class (either + 1 or -1). We rank these genes by F score and then select the top 25 gene sets as the features.

In contrast to the parametric approach, nonparametric approaches rank samples of each gene using their expression level and punish the disorders that damage a perfect sample split. The less the punishment, the smaller the score a gene receives. This means that a gene is more informative if it has a smaller corresponding score. In this study, we use three nonparametric methods: Threshold Number of Misclassification (TNoM), Minimum Distance to Modal Ranking (MDMR), and Weighted Punishment on Overlap (WEPO).

### Threshold Number of Misclassification (TNoM)

TNoM assumes that an informative gene has different values between the two classes, and thus we are able to separate these using a threshold value. A decision rule corresponding to a given expression level, such as sign (*ax + b*), is used to score the given gene and predict the unknown class. TNoM looks to select the values of *a *and *b *in order to minimize the number of errors:

We then rank these genes with a TNoM score and select the top 25 gene sets as the features.

### Minimum Distance to Modal Ranking (MDMR)

The MDMR method first ranks all the sample values of a gene and then computes the minimum distance between these ranks and a modal rank. The ranking algorithm, described by Park *et al *[28], is used in this study. A score is defined as the minimum number of consecutive swaps needed to arrive at a perfect split of two classes. A score of 0 represents the gene that can split two classes exactly. The MDMR score is defined as

where h(x) is the indicator function

We then rank these genes with an MDMR score and select the top 25 genes for the study.

### Weighted Punishment on Overlap (WEPO)

Chung et al. [30] proposed the WEPO method to reduce possible loss of information when using the TNoM or MDMR methods. Because genes with identical ordered expression data may not have the same discriminative power, WEPO introduces the z-score into the rank swapping scheme in order to avoid this problem. For gene *k*, the expression levels of samples are first normalized via z-score to eliminate the problem of scaling. The z-score is defined as

where *μ *is the sample mean and MAD is the mean absolute deviation of gene k. The punished score of each gene is calculated by estimating the overlapping regions of the two classes. The punishment is defined as

## Machine Learning-Based Gene Selection Methods

Identifying biologically relevant genes, such as cancer-related genes, from microarray gene expression data is one of the most important areas in modern medical research. In addition to the six statistical methods described in the previous section, we also propose two machine learning-based gene selection methods: Random Forest Gene Selection (RFGS) and Support Vector Sampling Technique (SVST).

### Random Forest Gene Selection (RFGS)

Random forest is an algorithm for classification developed by Leo Breiman [31] that uses an ensemble of classification trees. Each of the classification trees is built using a bootstrap sample of the data, and at each split the candidate set of variables is comprised of a random subset. Thus, random forest uses both bagging and random variable selection for tree building.

**Figure 1:**
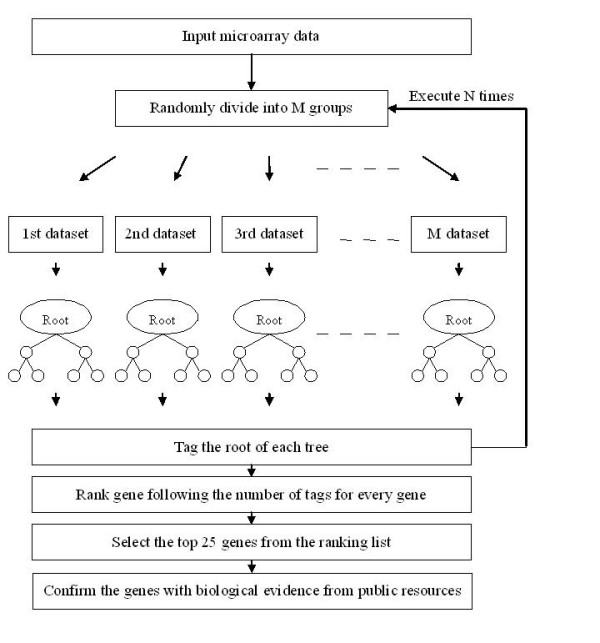
**The flowchart of the random forest gene selection method**.

In this paper, we propose a random forest concept to identify biologically relevant genes. The flowchart of our approach is shown in Figure 1. We first randomly divide all genes into 1000 groups; for example, there are approximately 7 genes in each group for the leukemia dataset and 13 genes in each group for the prostate cancer dataset. When all genes are randomly assigned into a group, we then build up a decision tree for each group. The most significant gene in each tree will serve as the root gene, and these root genes are marked by adding a number in the gene array. After the first cycle is completed, we initiate another cycle by again randomly assigning all genes, and this process is repeated for 100 cycles. The more frequently a gene is selected as the root, the higher a score it will receive. After 100 cycles, all genes will be ranked based on their score. In this paper, we select the top 25 genes and confirm them based on supporting evidence culled from current medical literature. If the genes are found to have a relationship with the target cancers, we call them "biologically relevant genes". Because the random forest approach may generate different biological genes each time, we run the code 10 times. Those genes which on average appear most consistently within the top 25 are used in comparison with the results of other methods.

This approach is displayed in the following pseudo code, where *X *is the cancer's gene expression data (containing *S *samples *G *and genes) and the *Y*^*S *^is the label of each sample.

The Pseudo Code of the Random Forest Gene Selection Method

*Input*: , *S *= 1.. *s*, *G *= 1.. *g*, *Y*^*S *^∈ {-1,1}, *X *∈ *Rg*

      s = number of samples, g = number of genes

Output: n top genes

1.   begin

2.   for i = 1 to S

3.      do normalize X

4.   end

5.   for I = 1 to N (N = 100 used here)

6.         while (All genes assigned completely)

7.            Randomly assign all genes into M groups (M = 1000 used here)

8.         for J = 1 to M

9.            Build up a decision tree on each group

10.            Mark the root of each group

11.         end

12.   end

13.   Rank gene following the number of marks for every gene

14.   Select the top 25 genes from the ranking list

15.   Confirm the genes with biological evidence from public resources

16.   Calculate the average biological genes found in the top 25 genes

### Support Vector Sampling Technique (SVST)

In the ongoing effort to improve the accuracy of cancer classification, many machine learning methods have been developed over the past few years. Among them, SVM is arguably one of the best methods. Although the SVM classification method has been widely used in the machine learning domain, there is little research focused on the actual support vectors. These support vectors have several computational and learning theoretic consequences [32]. Gene selection is a common way to avoid the high dimensional feature problem; however, the majority of past research has applied gene selection algorithms using all available samples. The accuracy of SVM is largely dependent on a hyperplane that can clearly separate different classes, and many samples may be outliers or may be separated incorrectly. Thus, using all samples could cause some degree of inaccuracy in classification performance.

In this paper, we develop a new method to identify biologically relevant genes using only quality samples which are located on support vectors. We assume that the use of support vectors is critical in eliminating irrelevant tissue composition-related genes. We called this method the support vector sampling technique (SVST). Our hypothesis is that by using samples located only on support vectors, we have a higher probability of identifying more relevant genes. To verify this hypothesis experimentally, we compared SVST with other statistical methods using two cancer datasets. SVST is a two-step process which includes first selecting support vector samples and then performing the SNR gene selection method. This approach allows us to narrow the field to only the most relevant samples in order to select the most biologically relevant genes.

The approach process is displayed in the following pseudo code. *X*is the cancer's gene expression data, containing *S *samples and *G *genes, and the *Y*^*S *^is the label of each sample.

The Pseudo Code of the SVST Method

*Input*: , *S *= 1.. *s*, *G *= 1.. *g*, *Y*^*S *^∈ {-1,1}, *X *∈ *Rg*

      s = number of samples, g = number of genes

Output: n top genes

1.   begin

2.   for i = 1 to S

3.      do normalize X

4.   end

5.   Set K = linear function

*6.   do train SVM*(*K*(*X*^*S*^), *Y*^*S*^) [6]

7.   sv = extract support vectors from training SVM

8.   for i = 1 to S

9.      svs = extract support vector samples by sv from all samples

10.   end

11.   for i = 1 to G

12.   r-genes = do SNR scoring function(svs)

13.   end

14.   rank r-genes by SNR score

15.     = select n top genes from r-genes

16.   end

### Theoretical basis of the SVST

The SVST is briefly described as follows. A binary SVM attempts to find a hyperplane which maximizes the "margin" between two classes (+ 1/-1). Let

be the gene expression data with positive and negative class labels, and the SVM learning algorithm should find a maximized separating hyperplane

where *W *is the n-dimensional vector (called the normal vector) that is perpendicular to the hyperplane, and *b *is the bias. The SVM decision function is shown in formula (1), where *α*_*i *_are positive real numbers and *ϕ *is the mapping function(1)

**Figure 2:**
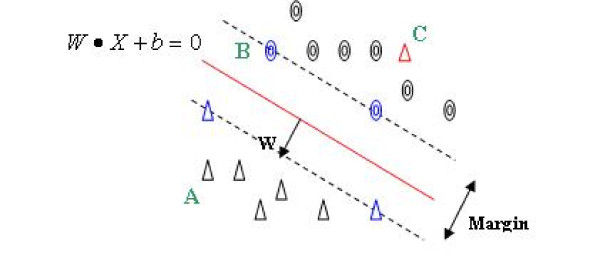
**The hyperplane and the support vectors**. The red line is the hyperplane which separates the two classes of samples. The blue points are the support vectors. A is the right classified sample but has less influence on the hyperplane. B is the right classified sample and has influence on the hyperplane. C is the incorrectly classified sample.

Only of *ϕ*(*X*_*i*_) of *α*_*i *_> 0 would be used, and these points are support vectors. The support vectors lie close to the separating hyperplane (shown in Figure 2). *α*_*i *_represents non-negative Lagrange multipliers, and it is used to discriminate every piece of training data which has a different influence on the hyperplane in high dimension feature spaces. To explain the meaning of *α*_*i*_, we first maximize the Lagrange problem:(2)

When *α*_*i *_= 0 then *L*_*D *_= 0 in formula (2), as in this case, *α*_*i *_means that the i th data has no influence on the hyperplane; therefore, this sample is correctly classified by the hyperplane (such as point A in Figure 2).

When 0 <*α*_*i *_< C, where C > 0 is the penalty parameter of the error term, the Lagrange problem *L*_*D *_is subject to

Therefore, *L*_*D *_= *α*_*i*_, and under this circumstance, *α*_*i *_means that the ith data has a degree of influence on the hyperplane (such as point B in Figure 2).

When *α*_*i *_= C, the Lagrange problem *L*_*D *_is subject to

*L*_*D *_is negative, and therefore, *α*_*i *_means the ith data is incorrectly classified by the hyperplane (such as point C in Figure 2). Each *α*_*i *_determines the degree by which each training example influences the SVM function. Because the majority of the training examples do not affect the SVM function, most of the *α*_*i *_are 0. We can then infer that these support vectors should contain the desired strong classification information. By extracting only the samples (such as point B) located on the hyperplane, we can run a gene selection algorithm that better identifies biologically relevant genes.

We applied our method to two microarray datasets for leukemia and prostate cancer. In order to simplify the selection of the SVM parameters, we tested several different settings to ascertain the best classification performance. The selection of SVM parameters used in our SVST method is summarized in Table 1. These parameter settings may not be optimized settings; however, they are sufficient for the selection of quality support vectors. Using these parameter values, we found 32 support vector samples in 72 leukemia samples and 44 support vector samples in 102 prostate cancer samples. We then used these samples to find the most informative genes through the SNR gene selection algorithm.

**Table 1:** Parameter settings in SVM for SVST method.

Parameter	Setting
Kernel Type	Linear

Gamma [Default: 1/(# of genes)]	1/7200 for leukemia 1/12600 for prostate cancer

Cost	1

## Results and Discussion

In this paper, we experiment using two cancer gene expression microarray datasets: leukemia and prostate cancer. We chose this data not only out of concern for the potential influence on human beings but also for the data's characteristics. Leukemia microarray data is easily classified; many cancer classification researchers consider this data as a performance comparison standard. Prostate cancer microarray data, however, is not easily classified. Therefore, utilizing both datasets provides a measurable way to demonstrate the benefits of our proposed methods.

### Application to the leukemia microarray dataset

#### Leukemia dataset

This original gene expression data was downloaded from http://www.genome.wi.mit.edu/MPR/[23]. The data contains 72 bone marrow or peripheral blood samples with either acute myeloid leukemia (AML) or acute lymphoblastic leukemia (ALL). The data set provides 7129 human genes produced by Affymetrix high-density olignucleotide microarrays. The intensity of gene expression is rescaled to normalize overall intensities for each microarray. Even though this data provides a plethora of genetic information, its feature dimension is too high for practical analysis. We need a selection method that can reduce this feature dimension.

#### Identifying biologically relevant leukemia genes

Table 2 compares the resulting biologically relevant genes in leukemia identified using the 8 methods. Among these 8 methods, WEPO finds the least number of biological genes at 5 genes, while TNoM identifies 6 genes. LSD and t-Test both identify 7 biological genes. SNR and random forest identify 8 and 9 biological genes respectively. SVST and MDMR find the most biologically relevant genes, where SVST identified 15 and MDMR identified 12. Our proposed SVST method has the best performance in terms of identifying biologically relevant genes for leukemia.

**Table 2:** The biologically relevant genes found in leukemia.

	SNR	t-TEST	LSD	TNoM	MDMR	WEPO	RFGS*	SVST
Gene1	ZYX	SNRPD1	SNRPD1	KLHDC10	ZYX	PTMA	MGST1	ZYX

Gene2	TCF3	PRPF18	LAMP2	BTG2	APLP2	CXCR4	CD63	TCF3

Gene3	CCND3	LAMP2	PRPF18	CD68	MGST1	IFITM3	SERPING1	CD33

Gene4	CST3	PRKCI	PRKCI	EIF4A1	CSTA	ADA	QSOX1	CD63

Gene5	CD33	MSH2	GTF2E2	PFKL	CD63	RPL23A	APLP2	TCRA

Gene6	CD79A	GTF2E2	MSH2	LIPE	CTSD		PLCB2	SPTAN1

Gene7	SPTAN1	DCK	ALCAM		LYN		POU2AF1	MPO

Gene8	Macmarcks				CLU		CTSD	CST3

Gene9					FAH		ACADM	HOXA9

Gene10					PLEK			CD79A

Gene11					MPO			Macmarcks

Gene12					LRPAP1			CCND3

Gene13								PSMB9

Gene14								IL18

Gene15								STOM

								

	8	7	7	6	12	5	9*	15

Comparison of biologically relevant genes in leukemia identified using 8 methods An * indicates the average number of biologically relevant genes found in the top 25 genes using the random forest gene selection method.

**Figure 3:**
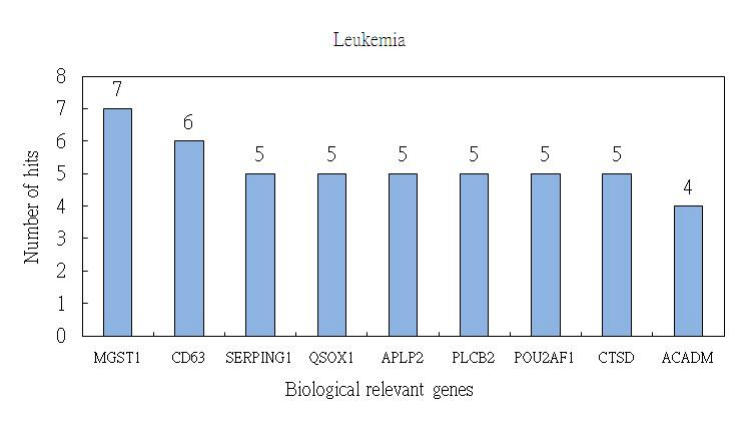
**The average number of hits generated by the random forest method regarding biologically relevant genes in leukemia**.

For the random forest method, we can identify, on average, 9 biologically relevant genes from the top 25 ranked genes. In Table 2, we show the results of running the method 10 times and order the relevant genes by decreasing number of hits. As shown in Figure 3, 9 genes are recorded in the following order: *MGST1 *(7 hits), *CD63 *(6 hits), *SERPING1 *(5 hits), *QSOX1 *(5 hits), *APLP2 *(5 hits), *PLCB2 *(5 hits), *POU2AF1 *(5 hits), *CTSD *(5 hits), and *ACADM *(4 hits).

Our proposed SVST method has the benefit of identifying more biologically relevant genes. For example, 15 genes (60%) were found to be biologically relevant to leukemia among the top 25 ranked genes selected using the SVST method. Table 3 describes the gene names and their possible function. The medical literature regarding each gene is included in the same table.

**Table 3:** Functions of the biologically relevant genes found in leukemia.

Gene Name	Gene Function	Evidence References
ZYX	Adhesion plaque protein. Binds alpha-actinin and the CRP protein. May be a component of a signal transduction pathway that mediates adhesion-stimulated changes in gene expression.	[36]

TCF3	Heterodimers between TCF3 and tissue-specific basic helix-loop-helix (bHLH) proteins play major roles in determining tissue-specific cell fate during embryogenesis, like muscle or early B-cell differentiation. Binds to the kappa-E2 site in the kappa immunoglobulin gene enhancer.	[37]

CD33	In the immune response, may act as an inhibitory receptor upon ligand induced tyrosine phosphorylation by recruiting cytoplasmic phosphatase(s).	[35]

CD63	This antigen is associated with early stages of melanoma tumor progression. May play a role in growth regulation. Lysosome membrane; Multi-pass membrane protein. Late endosome membrane; Multi-pass membrane protein. Note = Also found in Weibel-Palade bodies of endothelial cells. Located in platelet dense granules. melanomas, hematopoietic cells, tissue macrophages.	[38]

TCRA	T cell receptor alpha-chain.	[39]

SPTAN1	Fodrin, which seems to be involved in secretion, interacts with calmodulin in a calcium-dependent manner.	[40]

MPO	Part of the host defense system of polymorphonuclear leukocytes. It is responsible for microbicidal activity against a wide range of organisms.	[41]

CST3	As an inhibitor of cysteine proteinases, this protein is thought to serve an important physiological role as a local regulator of this enzyme activity.	[42]

HoxA9	Sequence-specific transcription factor which is part of a developmental regulatory system that provides cells with specific positional identities on the anterior-posterior axis.	[33]

CD79A	Required in cooperation with CD79B for initiation of the signal transduction cascade activated by binding of antigen to the B-cell antigen receptor complex.	[43]

Macmarcks	May be involved in coupling the protein kinase C and calmodulin signal transduction systems.	[34]

CCND3	Essential for the control of the cell cycle at the G1/S (start) transition. Potentiates the transcriptional activity of ATF5.	[44]

PSMB9	The proteasome is a multicatalytic proteinase complex which is characterized by its ability to cleave peptides with Arg, Phe, Tyr, Leu, and Glu adjacent to the leaving group at neutral or slightly basic pH. The proteasome has an ATP-dependent proteolytic activity. This subunit is involved in antigen processing to generate class I binding peptides.	[26]

IL18	Augments natural killer cell activity in spleen cells and stimulates interferon gamma production in T-helper type I cells.	[45]

STOM	Interacting selectively with one or more specific sites on a receptor molecule, a macromolecule that undergoes combination with a hormone, neurotransmitter, drug or intracellular messenger to initiate a change in cell function.	[46]

The 15 biologically relevant genes found in the top 25 ranked genes in leukemia selected using the SVST method.

In this section, we individually examine these 15 genes for relevance in the diagnosis of leukemia. All 15 genes have some relevance to leukemia and deserve a more detailed analysis to understand their role in the cancer's development. The role of some of these biologically relevant genes can be easily explained because they code for proteins whose role in leukemia has been long identified and widely studied. Such is the case of the HoxA9 gene, where ***Hoxa9 ***collaborates with other genes to produce highly aggressive acute leukemic disease [33]. The other example is the Macmarcks gene, where tumor necrosis factor-alpha rapidly stimulates ***Macmarcks ***gene transcription in human promyelocytic leukemia cells [34]. The presence of some of the other genes in our list can be explained by recently published studies. For example, the role of the CD33 gene, ***CD33***, is a myeloid cell surface antigen that is expressed on blast cells in acute myeloid leukemia (AML) in a majority of all patients regardless of age or subtype of disease [35].

The role of the 15 genes in Table 3 is described as follows. The ZYX gene: ***Zyxin ***encodes a LIM domain protein localized at focal contacts in adherent erythroleukemia cells [36]. The TCF3 gene: The t(1;19)(q23;p13.3) is one of the most common chromosomal abnormalities in B-cell precursor acute lymphoblastic leukemia and usually gives rise to the ***TCF3-PBX1 ***fusion gene. The ***TCF3 ***gene has been shown to be involved in the majority of cases with a cytogenetically visible t(1;19) translocation, while the remaining TCF3-negative ALLs demonstrated breakpoint heterogeneity [37]. The CD63 gene: In the rat basophilic leukemia cell line, an antibody against CD63 (AD1) inhibited immunoglobulin E (IgE)-mediated histamine release, suggesting a role for CD63 in events associated with mediator release [38]. The TCRA gene: T-cell prolymphocytic leukemia is a sporadic, mature T-cell disorder in which there is usually an aberrant T-cell receptor alpha (***TCRA***) rearrangement that activates the TCL1 or MTCP1-B1 oncogenes [39]. The ***SPTAN1 ***gene: In a human chronic myelogenous leukemia cell line with the Ph1 chromosome, K562, the SPTAN1 mapped centromeric to the translocation breakpoint, indicating that the alpha-fodrin gene is not translocated to the Ph1 chromosome in this cell line [40]. The ***MPO ***gene: The tumour cells were positive for CD68 (KP1), CD68 (PGM1), lysozyme and CD45. They were negative for MPO, CD15, CD163, TdT, CD117, T and B cell markers [41]. The CST3 gene: Sun Y explores differentially expressed genes in leukemia gene expression profiles and identifies main related genes in acute leukemia. The results show that in four patient/donor pairs with ALL, 5 up-regulated (RIZ, STK-1, T-cell leukemia/lymphoma 1A, Cbp/p300, Op18) and 1 down-regulated genes (hematopoietic proteoglycan core protein) were identified. In five patient/donor pairs with AML, 6 up-regulated (STAT5B, ligand p62 for the Lck SH2, ***CST3***, LTC4S, myeloid leukemia factor 2 and ***epb72***) and 1 down-regulated genes (CCR5) were identified [42]. The ***CD79A ***gene: Expression of the ***CD79A ***(MB-1) chain has been studied in leukemia and is shown to be present in most B lineage acute lymphoblastic leukemia [43]. The CCND3 gene: A 51-bp deletion was detected in ***CCND3 ***in a patient with normal karyotype acute myeloid leukemia [44]. The PSMB9 gene: ***PSMB9 (LMP2) ***is expressed both in normal EBV latency and EBV-associated pathologies. EBV is associated with a variety of haematopoietic cancers such as African Burkitt's lymphoma, Hodgkin's, and adult T-cell leukemia [26]. The IL18 gene: ***IL18 (IGIF) ***proposed to be designated as IL-18, selectively up-regulates ICAM-1 expression in KG-1 cells, a human myelomonocytic cell line, human IL-18 was measurable in the plasma of leukemia patients [45]. The STOM gene: STORP is homologous to the ***STOM (Epb72) ***gene coding for the erythrocyte band 7 integral membrane proteins or stomatin. The STORP gene is positioned 2 kb upstream of the promyelocytic leukemia gene in a head-to-head configuration [46].

### Application to the prostate cancer microarray dataset

#### Prostate cancer dataset

The original gene expression data for prostate cancers is available at http://www.genome.wi.mit.edu/cgi-bin/cancer/datasets.cgi[47]. The dataset contains expression levels for 52 prostate tumor samples and 50 normal samples. Each sample contains 12600 genes measured using Afffymertix oligonucleotide arrays. We set the tumor sample to (-1) and the normal samples to (+ 1), and we then merged these data sets together for the 8 methods.

#### Identifying biologically relevant prostate cancer genes

To complete our study, we proceed similarly with the prostate cancer data by running our 8 gene selection methods on the entire dataset of 102 samples. The comparison of biologically relevant genes in prostate cancer identified using these 8 methods are shown in Table 4. Among these 8 methods, TNoM finds the least number of genes at 3 genes. The MDMR and WEPO methods identify 7 genes and 8 genes respectively. All the following four methods (SNR, LSD, t-Test, and Random forest) are in the next group, where they identify 9 biologically relevant genes. SVST method, once again, is capable of finding the most at 13 biologically relevant genes. Among these 8 methods, our proposed SVST method has the best performance.

**Table 4:** The biologically relevant genes found in prostate cancer.

	SNR	t-Test	LSD	TNoM	MDMR	WEPO	RFGS*	SVST
Gene1	HPN	UCK2	UCK2	NFIX	HPN	NF2	PTGDS	HPN

Gene2	PTGDS	LPIN1	LPIN1	FOXG1	PDIA5	PTGDS	HPN	NELL2

Gene3	NELL2	KIAA0746	KIAA0746	PML	ICA1	KLK3	CLU	PTGDS

Gene4	S100A4	GNB2L1	GNB2L1		AGR2	CLU	NELL2	S100A4

Gene5	TARP	CAV2	CAV2		KLK3	MYL6	SERPINF1	TNFSF10

Gene6	COL4A6	IGBP1	IGBP1		UAP1	FLNA	HSPA8	SERBP1

Gene7	ANGPT1	CASP3	CASP3		FBP1	SERPING1	XBP1	RBP1

Gene8	RBP1	DOPEY2	DOPEY2			ACTG2	ALCAM	GSTM1

Gene9	GSTM1	PDIA5	PDIA5				AGR2	ANGPT1

Gene10								LMO3

Gene11								COL4A6

Gene12								DIO2

Gene13								TARP

								

	9	9	9	3	7	8	9*	13

Comparison of biologically relevant genes in prostate cancer identified using 8 methods. An * indicates the average number of biologically relevant genes found in the top 25 genes using the random forest gene selection method.

**Figure 4:**
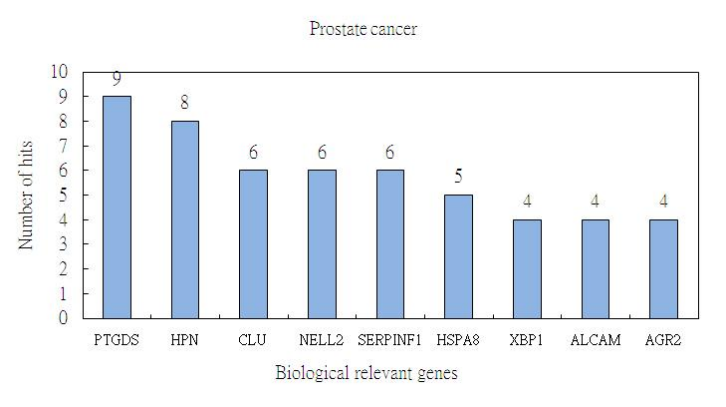
**The average number of hits generated by the random forest method regarding biologically relevant genes in prostate cancer**.

For the random forest method, we identify, on average, 9 biologically relevant genes in the top 25 ranked genes. In Table 2, we show the results gathered from running the method 10 times and order the biologically relevant genes by decreasing of number of hits. As shown in Figure 4, these 9 genes are recorded in the following order: *PTGDS *(9 hits), *HPN *(8 hits), *CLU *(6 hits), *NELL2 *(6 hits), *SERPINF1 *(6 hits), *HSPA8 *(5 hits), *XBP1 *(4 hits), *ALCAM *(4 hits), and *AGR2 *(4 hits).

Table 5 lists 13 genes found to be biologically relevant to prostate cancer among the top 25 ranked genes selected using the SVST method. The possible function of each gene and its medical references are also included in Table 5. In this section, we individually examine these 13 top ranked genes for relevance in the diagnosis of prostate cancer. All 13 genes have some relevance and deserve a more detailed analysis to understand their role in prostate cancer's development. The role of some of these biologically relevant genes can be easily explained because they code for proteins whose role in prostate cancer has long been identified and widely studied. Such is the case of the NELL2 gene. In situ hybridization analysis of hyperplastic prostate specimens demonstrated that ***NELL2 ***mRNA expression is predominantly localized in basal cells of the epithelium. Disease-related changes in the levels of ***NELL2 ***may contribute to alterations in epithelial-stromal homeostasis in BPH [48]. The presence of some other genes in our list can be explained by recently published studies. For example, the discovery that the GSTM1 gene, ***GSTM1***, may be linked to prostate cancer risk was published only a year ago [49]. Another example is the ANGPT1 gene, published 2 years ago, where Ang-2 was expressed predominantly in prostate epithelial cells whereas Ang-1 (***ANGPT1***) was expressed in epithelium and smooth muscle [50].

**Table 5:** Functions of the biologically relevant genes found in prostate cancer.

Gene Name	Gene Function	Evidence References
HPN	Plays an essential role in cell growth and maintenance of cell morphology.	[59]

S100A4	S100 calcium binding protein A4.	[52]

RBP1	Intracellular transport of retinol.	[25]

ANGPT1	Appears to play a crucial role in mediating reciprocal interactions between the endothelium and surrounding matrix and mesenchyme.	[50]

COL4A6	Type IV collagen is the major structural component of glomerular basement membranes (GBM), forming a 'chicken-wire' meshwork together with laminins, proteoglycans, and entactin/nidogen.	[53]

NELL2	Chicken nel-like 2 homolog with a wide and weak expression, expressed in adult and fetal brain and hemopoietic cells (nucleated peripheral blood cells) but not in B cells.	[48]

GSTM1	Conjugation of reduced glutathione to a wide number of exogenous and endogenous hydrophobic electrophiles.	[49]

PTGDS	It is likely to play important roles in both maturation and maintenance of the central nervous system and male reproductive system.	[54]

TARP	Transmembrane receptor activity.	[58]

LMO3	Lim domain only 3.	[56]

DIO2	Essential for providing the brain with appropriate levels of T3 (3,5,3'-triiodothyronine) during the critical period of development.	[57]

SERBP1	May play a role in the regulation of mRNA stability.	[55]

TNFSF10	Induces apoptosis. Its activity may be modulated by binding to the decoy receptors TNFRSF10C/TRAILR3, TNFRSF10D/TRAILR4 and TNFRSF11B/OPG that cannot induce apoptosis.	[51]

We also list the roles of the rest of the biological genes shown in Table 5. The TNFSF10 gene: the FOXO family of forkhead transcription factors is implicated in ***TNFSF10 ***transcriptional activation in prostate carcinoma cells [51]. The S100A4 gene: ***S100A4 ***protein is expressed in neither benign nor malignant prostatic epithelium nor in LNCaP and Du145 cells. The mechanism underlying absent ***S100A4 ***expression in prostatic epithelium and cell lines may involve methylation [52]. The RBP1 gene: Altered ***CRBP1 ***expression and promoter hypermethylation occur in several tumours, these changes were investigated in prostate tumorigenesis [25]. The COL4A6 gene: ***COL4A6 ***expression is missing in nearly all cancerous tissues as evidenced by the Boolean function [53]. The PTGDS gene: Lipocalin-type prostaglandin D syntheses (***L-PGDS***) and prostaglandin D2 (***PGD2***) metabolites produced by normal prostate stromal cells inhibited tumor cell growth through a peroxisome proliferator-activated receptor gamma (PPARgamma)-dependent mechanism [54]. The SERBP1 gene: The expression of hepsin, uPA, PAI-RBP1 (***SERBP1***), PAI-1, and factor XIII may influence fibrinolysis and are regulated by the tumour microenvironment [55]. The LMO3 gene: The protein encoded in this gene is a LIM-only protein (***LMO***), which is involved in cell fate determination. This gene has been noted to up-regulate in the prostate cancer samples [56]. The DIO2 gene: Subtype II tumours represent the second clinically aggressive tumour subclass, and the gene expression feature that characterizes this subgroup includes several genes identified in supervised analysis to be associated with both high grade and advanced stage cancer, such as HDAC9 and ***DIO2 ***[57]. The ***TARP ***gene: TARP is exclusively expressed in the prostate in males and is up-regulated by androgen in LNCaP cells, an androgen-sensitive prostate cancer cell line [58]. The HPN gene: Xu L has identified a pair of robust marker genes (***HPN ***and STAT6) by integrating microarray datasets from three different prostate cancer studies [59].

In Table 6, we summarize the results from related studies. Since few studies focus on identifying biologically relevant genes in cancers, we summarize their results based on the study's computing methods. We then compare all these 8 gene selection methods, including our two proposed novel methods, with the results in Table 6. In different cancer types and methods, very few biologically relevant genes are identified. Our methods, especially our proposed SVST method, are significantly superior to these. For example, in the leukemia dataset, the SVST method identifies 15 biologically relevant genes out of the top 25 while Covell et al. [60] identifies 11 biologically relevant genes out of the top 68. In the prostate cancer dataset, the SVST method identifies 13 biologically relevant genes out of the top 25 while Covell et al. [60] identifies only 6 biologically relevant genes out of the top 36.

**Table 6:** Comparison of related methods and results.

Authors	Methods	Cancer Type	Results
Ben-Tor et al. [27]	TNoM	Ovarian	4/137 (Among the top 137 genes, 8 are cancer-related genes. 4 genes (GAPDH, SLPI, HE4 and keratin 18) are ovarian genes.)

Covell et al. [60]	SOM	Bladder	1/5 (1 out of the top 5 genes is a Bladder gene)
	Up-regulated in tumor cells and down-regulated in normal cells	Breast	1/3 (1 out of the top 3 genes is a Breast gene)
		CNS	5/62 (5 out of the top 62 genes are CNS genes)
		Colorectal	2/37 (2 out of the top 37 genes are Colorectal genes)
		Leukemia	11/68 (11 out of the top 68 genes are Leukemia genes)
		Lung	1/4 (1 out of the top 4 genes is a Lung gene)
		Lymphoma	7/33 (7 out of the top 33 genes are Lymphoma genes)
		Melanoma	3/12 (3 out of the top 12 genes are melanoma genes)
		Mesothelioma	0/49 (0 out of the top 49 genes is a Mesothelioma gene)
		Pancreas	2/9 (2 out of the top 9 genes are Pancreas genes)
		Prostate	6/36 (6 out of the top 36 genes are Prostate genes)
		Renal	4/26 (4 out of the top 26 genes are Renal genes)
		Uterine	1/42 (1 out of the top 42 genes is a Uterine gene)

#### Statistically sound performance comparison among these 8 methods

As Ambroise and McLachlan [61] point out, the performance of a classification method may be overestimated when using the Leave-out-out method. In this study, therefore, we verified our experiment using a random average 3-fold method. This method randomly separates datasets into 3-folds and chooses one subset among the three as the validation set used to verify the model. The remaining two subsets are used as the model's training sets. The cross validation process is repeated 3 times with each of the three subsets used once for validation. This process is then repeated 100 times in order to gain a statistically impartial performance result for our model. In order to compare the classification performance of the 8 methods used in the paper, we used the SVM classifier with the linear kernel function and with default parameter settings.

**Figure 5:**
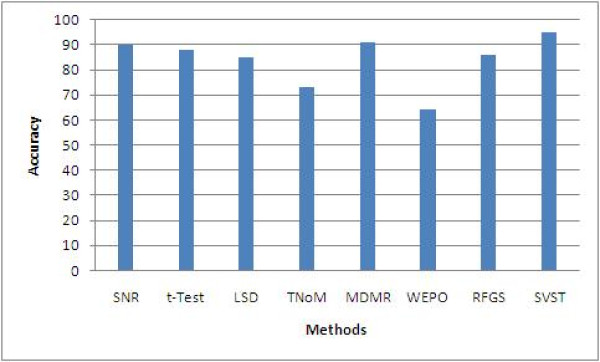
**Statistically sound performance comparison among 8 methods for the leukemia dataset**.

The performance comparison of these 8 methods is summarized in Table 7. This table shows the average classification accuracy (with ranges in the parentheses) after running experiments for all 8 methods 100 times as discussed in this paper. For ease of visualization, we also drew a bar comparison as shown in Figure 5. The results clearly show the superior performance of our SVST method. Compared to the other 7 methods, SVST significantly improves upon the average classification accuracy rate from 5% to 30% for leukemia datasets.

**Table 7:** Statistically sound performance comparison for the leukemia dataset.

Methods	25 genes	50 genes	75 genes	100 genes	125 genes	150 genes
SNR	.90(.87 to 1)	.93(.87 to .99)	.94(.89 to .1)	.95(.87 to .99)	.94(.88 to 1)	.96(.85 to 1)

t-Test	.88(.67 to 1)	.91(.66 to .99)	.91(.69 to .99)	.91(.65 to 1)	.92(.69 to .99)	.92(.64 to 1)

LSD	.85(.50 to 1)	.88(.53 to .95)	.89(.51 to .94)	.89(.52 to 1)	.87(.54 to .97)	.89(.54 to 1)

TNoM	.73(.67 to .91)	.73(.65 to .90)	.73(.66 to .91)	.73(.67 to .90)	.76(.69 to .92)	.75(.67 to .92)

MDMR	.91(.79 to 1)	.93(.74 to .98)	.93(.72 to .96)	.94(.78 to 98)	.94(.76 to .1)	.94(.79 to .99)

WEPO	.64(.46 to .79)	.61(.51 to .79)	.60(.50 to 76)	.67(.52 to 81)	.69(.50 to .85)	.73(.53 to .86)

RFGS	.86(.75 to .95)	.85(.76 to .98)	.85(.75 to .94)	.86(.75 to .95)	.88(.78 to .99)	.86(.73 to .97)

SVST	.95(.88 to 1)	.98(.87 to .99)	.97(.85 to .1)	.98(.87 to 1)	.98(.88 to .99)	.97(.87 to 1)

**Figure 6:**
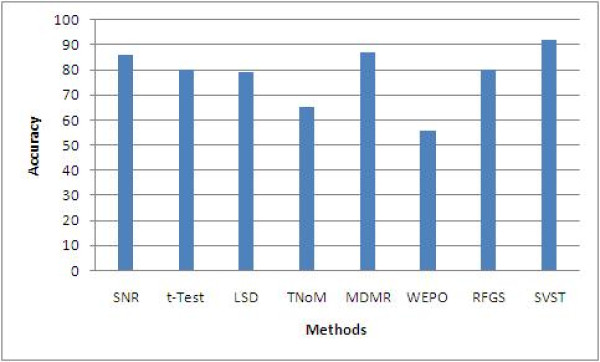
**Statistically sound performance comparison among 8 methods for the prostate cancer dataset**.

The same approach is also applied to prostate cancer datasets. Table 8 summarizes the performance comparison of 8 methods for analyzing prostate cancer datasets. A bar comparison is also shown in Figure 6. Once again, our SVST method demonstrates significant improvement in classification performance compared to 7 alternative methods. The average classification accuracy rate increased from 5% to 35% for prostate datasets. The results imply that the genes selected using the SVST not only are more biologically relevant but are also more informative with regards to the diagnosis and treatment of both leukemia and prostate cancers.

**Table 8:** Statistically sound performance comparison for the prostate cancer dataset.

Methods	25 genes	50 genes	75 genes	100 genes	125 genes	150 genes
SNR	.86(.82 to .95)	.86(.82 to .95)	.85(.80 to .97)	.86(.83 to .95)	.83(.80 to .93)	.84(.82 to .96)

t-Test	.80(.67 to .94)	.82(.66 to .92)	.82(.67 to .90)	.81(.67 to .93)	.81(.68 to .93)	.80(.69 to .95)

LSD	.79(.65 to .94)	.81(.63 to .93)	.81(.62 to .95)	.81(.64 to .95)	.81(.67 to .94)	.82(.64 to .93)

TNoM	.65(.53 to .80)	.65(.51 to .78)	.63(.50 to .79)	.65(.53 to .80)	.65(.52 to .78)	.63(.51 to .81)

MDMR	.87(.76 to .95)	.84(.75 to .97)	.86(.76 to .98)	.86(.75 to .97)	.87(.78 to .95)	.87(.74 to .98)

WEPO	.56(.43 to .70)	.57(.44 to .69)	.67(.53 to .74)	.70(.55 to .79)	.68(.52 to .75)	.73(.64 to .86)

RFGS	.80(.65 to .91)	.81(.68 to .92)	.78(.63 to .91)	.82(.68 to .92)	.79(.65 to .90)	.81(.67 to .92)

SVST	.92(.85 to .95)	.90(.83 to .96)	.91(.84 to .95)	.92(.87 to .94)	.92(.82 to .95)	.93(.81 to .97)

#### Preliminary study of gene-gene interaction of biologically relevant leukemia genes identified by the SVST method

Due to the superior characteristics of our SVST method (i.e. identifying a greater number of biologically relevant genes and yielding better classification accuracy rates), we would like to further investigate the possible gene-gene interactions among these biologically relevant genes. Our hypothesis is that the gene-gene interactions among these biologically relevant genes, if present, may provide additional benefits with regards to the diagnosis of cancers. As a preliminary study, we ran the experiment using 15 biologically relevant genes selected from a leukemia dataset. At first, we screened several protein-protein interaction (PPI) websites, and we found the IPIR (integrated protein interaction resource, http://ymbc.ym.edu.tw/ipir/) to be an excellent tool for building PPI graphs of leukemia gene products. The IPIR is a powerful web tool which retrieves protein-protein interaction information from BIND, DIP, HPRD, MINT, MIPS, and IntAct databases.

**Figure 7:**
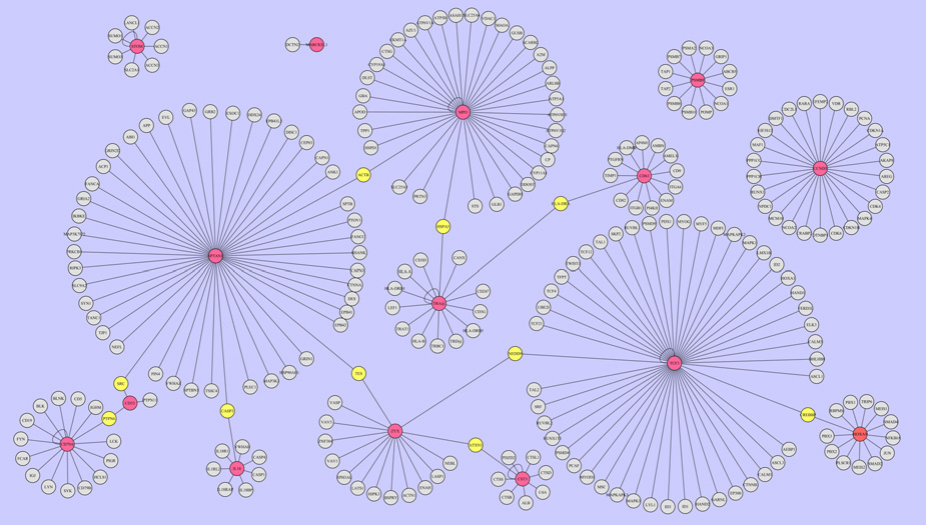
**The gene-gene interaction graph of biologically relevant leukemia genes identified by the SVST method**.

The protein-protein interaction graph based on the 15 biologically relevant leukemia genes selected using the SVST method is displayed in Figure 7. The name of each gene is viewable by expanding the scale of graph. In Figure 7, we find that 11 biologically relevant genes (marked in red) out of 15 genes form a PPI graph. To further understand the relationships among these genes, we summarize the neighbourhood genes and bridge genes in Table 9. The 11 biologically relevant genes we found which have interactions are ZYX, TCF3, CD33, CD63, TCRA, SPTAN1, MPO, CST3, HOXA9, CD79A, and IL18. Among these 11 genes, TCF3 has the largest number of interacting genes (47). SPTAN1 has the second largest number of interacted genes (46), and the remaining 9 genes (with the number of interacting genes shown in the subsequent parentheses) are: MPO (35), CD79A (16), ZYX (15), TCRA (15), CD63 (13), HOXA9 (13), CST3 (9), IL18 (8), and CD33 (3).

**Table 9:** The gene-gene interaction among identified leukemia genes.

Biologically relevant gene1	Number of interacted gene	Bridge gene between gene1 and gene2	Biologically relevant gene2
ZYX	15	NEDD9	TCF3

		ATXN1	CST3

		TES	SPTAN1

TCF3	47	NEDD9	ZYX

		CREBBP	HOXA9

CD33	3	PTPN6	CD79A

		SRC	SPTAN1

CD63	13	HLADRA	TCRA

TCRA	15	HLADRA	CD63

		HSPA5	MPO

SPTAN1	46	ACTB	MPO

		CASP3	IL18

		TES	ZYX

		SRC	CD33

MPO	35	ACTB	SPTAN1

		HSPA5	TCRA

CST3	9	ATXN1	ZYX

HOXA9	13	CREBBP	TCF3

CD79A	16	PTPN6	CD33

MACMARCKS	1		-

CCND3	26		-

PSMB9	13		-

IL18	8	CASP3	SPTAN1

STOM	8	-	

Preliminary study of gene-gene interaction of biologically relevant leukemia genes identified by the SVST method

There are several sub-networks among these genes. For instance, the sun-network links ZYX with TCF3, CST3, and SPTAN1via NEDD9, ATXN1, and TES, respectively (marked in yellow). The sun-network links TCF3 with ZYX and HOXA9 via NEDD9 and CREBBP, respectively. The sun-network links CD33 with CD79A and APTAN1 via PTPN6 and SRC, respectively. The sun-network links CD63 with TCRA via HLADRA. The sun-network links TCRA with CD63 and MPO via HLADRA and HSPA5, respectively. The sun-network links SPTAN1 with MPO, IL18, ZYX, and CD33 via ACTB, CASP3, TES, and SRC, respectively. The sun-network links MPO with SPTAN1 and TCRA via ACTB and HSPA5, respectively. The sun-network links CST3 with ZYX via ATXN1. The sun-network links HOXA9 with TCF3 via CREBBP. The sun-network links CD79A with CD33 via PTPN6. The sun-network links IL18 with SPTAN1 via CASP3.

Whether the identified PPI graph is the key mechanism to better classification performance currently remains unproven and is beyond the scope of this particular paper. However, our SVST method has the capability to identify a group of biologically relevant leukemia genes with a significant gene-gene interaction relationship. We believe this finding merits further study.

## Conclusions

It is difficult in cancer research to identify sensitive and specific gene markers. In order to overcome problems caused by high dimensional input spaces, accurate and efficient gene selection methods are critical. Traditional selection approaches, however, do not consider the quality of the samples they analyze, the result of which affects the selection of biologically relevant genes.

In this paper, we have proposed two novel gene selection algorithms, the SVST and the RFGS methods. Both identify more biologically relevant genes concerning leukemia and prostate cancer. The proposed RFGS method is capable of searching for a global optimal or near optimal subset of genes due to their randomized characteristics. The proposed SVST method first extracts quality samples (i.e. support vector samples located only on support vectors) and avoids selecting incorrect genes. These quality samples are then used to form an optimal subset of genes that have a better chance to be biologically relevant.

We demonstrate experimentally that our proposed RFGS and SVST methods identify more genes relevant to cancers. Our proposed RFGS method has the ability to identify an average of 9 biologically relevant genes out of the top 25 genes in both leukemia and prostate cancers. Our proposed SVST method produces the best results among all 8 methods. From the top 25 genes selected using SVST method, we find that 15 are biologically relevant in patients with leukemia and 13 genes are biologically relevant in patients with prostate cancers. In contrast to traditional statistical methods, which only identify 8 or less genes in patients with leukemia and 8 or less genes in patients with prostate cancer, our methods yield significantly better results. The significance of identifying biologically relevant genes cannot be understated; research in the fields of biology and medicine can benefit substantially from the identification of biologically relevant genes to confirm recent discoveries in cancer research or suggest new avenues for exploration.

## Authors' contributions

AHC initialed the study, designed the computational experiments, validated the results, drafted the manuscript, and obtained funding. YWT and CHL were involved in data acquisition, codes writing and testing. All authors were involved in data analysis and critical revision of the manuscript. All authors read and approved the final manuscript.
